# How Does the Cause of Infantile Hemiparesis Influence Other Conditioning Factors? A Preliminary Study in a Spanish Population

**DOI:** 10.3390/children8050323

**Published:** 2021-04-22

**Authors:** Rocío Palomo-Carrión, Rita Pilar Romero-Galisteo, Helena Romay-Barrero, Inés Martínez-Galán, Cristina Lirio-Romero, Elena Pinero-Pinto

**Affiliations:** 1 Department of Nursery, Physiotherapy and Occupational Therapy, Faculty of Physiotherapy and Nursery, University of Castilla-La Mancha, 45071 Toledo, Spain; Rocio.Palomo@uclm.es (R.P.-C.); Helena.Romay@uclm.es (H.R.-B.); Ines.Martinez@uclm.es (I.M.-G.); Cristina.Lirio@uclm.es (C.L.-R.); 2 GIFTO, Group of Research in Physiotherapy of Toledo, 45071 Toledo, Spain; 3 Department of Physiotherapy, Faculty of Health Sciences, University of Málaga, 29016 Málaga, Spain; 4 Department of Physiotherapy, Faculty of Nursery, Physiotherapy and Podiatry, University of Seville, 41009 Seville, Spain; epinero@us.es

**Keywords:** associated disorders, early diagnosis, early intervention, family, infantile hemiparesis, stroke cause

## Abstract

Infantile hemiparesis may be associated with significant morbidity and may have a profound impact on a child’s physical and social development. Infantile hemiparesis is associated with motor dysfunction as well as additional neurologic impairments, including sensory loss, mental retardation, epilepsy, and vision, hearing, or speech impairments. The objective of this study was to analyze the association between the cause of infantile hemiparesis and birth (gestational age), age of diagnosis, and associated disorders present in children with infantile hemiparesis aged 0 to 3 years. An observational and cross-sectional study was performed. A simple and anonymous questionnaire was created ad hoc for parents of children diagnosed with infantile hemiparesis aged between 0 and 3 years about the situation regarding the diagnosis of hemiparesis, birth, cause of hemiparesis, and presence of other associated disorders. Perinatal stroke (60.1%) was the most common cause of hemiparesis, and the most typical associated disorder was epilepsy (34.2%), with the second largest percentage in this dimension corresponding to an absence of associated disorders (20.7%). The most frequent birth was “no premature” (74.1%). The mean age of diagnosis of infantile hemiparesis was registered at 8 months (IQR: 0–36). Knowing the possible association between different conditioning factors and the cause of infantile hemiparesis facilitates the prevention of severe sequelae in children and family, implementing an early comprehensive therapeutic approach in children with infantile hemiparesis.

## 1. Introduction

Infantile hemiparesis may be associated with significant morbidity and may have a profound impact on the child’s physical and social development [1]. Additionally, infantile hemiparesis is associated with motor dysfunction as well as additional neurologic impairments, including sensory loss, mental retardation, epilepsy, and vision, hearing, and speech impairments [2,3]. The increasing percentage of low birth weight and prematurity is connected to all subtypes of cerebral palsy (CP) [4]. In these infants, the type of brain injury associated with CP is primarily periventricular leukomalacia and intraventricular hemorrhage [4,5]. It seems that the spastic hemiplegic subtype is the most common clinical form of CP in both term (>37 weeks) and premature infants (<37 weeks) [6,7,8]. Throughout the years, the cause of hemiparesis had remained quite heterogenic, with possible involvement of a wide range of mechanisms in both term and premature infants [9]. Perinatal stroke is defined as the commonest cause of infantile hemiparesis [10]. Its etiology is multifactorial, and the risk increases when multiple risk factors are present [11], such as the high incidence of intrapartum risk factors in term infants, present in 68%, 76%, and 85% of infants according to different studies [12,13].

The period of diagnosis can be very confusing. Due to variability in the motor results and the probable comorbidity with other health complications, in many cases, there is a lack of understanding of the impact and future implications when the diagnosis is established [13]. During this time, parents begin to face the challenges of CP, reporting greater stress and lack of resources than parents of older children with the same pathology [14]. Early detection and diagnosis of CP are essential to initiate early intervention as soon as possible and optimize the use of resources [15]. After diagnosis, families will have the opportunity to search for information about the diagnosis and prognosis of the condition and how to choose the most adequate intervention for their child and for themselves [16]. On the other hand, delays in diagnosis can have negative consequences in the long term for both the child and the family [17]. Therefore, common efforts should be targeted at identifying recommendations based on evidence for the diagnosis and management of CP. Moreover, as is recommended by clinical studies, parents must be part of both processes, that is, early diagnosis and intervention [18].

Thus, the main objective of this study was to analyze the association between the cause of infantile hemiparesis and the gestational age, age of diagnosis, and associated disorders present in children with infantile hemiparesis aged 0 to 3 years.

## 2. Materials and Methods

### 2.1. Study Design

Observational and cross-sectional study.

### 2.2. Participants

The study was composed of 193 families resident in Spain, whose children had been diagnosed with infantile hemiparesis and were aged 0–3 years.

### 2.3. Inclusion/Exclusion Criteria

To complete the questionnaire and be included in the dataset, the participants were required to: have residence in Spain for a minimum of 3 years before completing the questionnaire and have a child diagnosed with infantile hemiparesis aged between 0 and 3 years.

The study excluded parents whose children had been diagnosed with a different type of CP that was not exclusively hemiparesis.

### 2.4. Instrument

A simple questionnaire was created ad hoc for parents of children diagnosed with infantile hemiparesis aged between 0 and 3 years (Appendix A).

The nine questions were designed with the aim of gathering information about the situation regarding the diagnosis of hemiparesis, birth, cause of hemiparesis, and presence of other associated disorders. This condition was selected for being one of the most frequent types and because certain aspects of it that affect the quality of life of the patients are still unknown.

The questionnaire was developed by a research team with a mother with 10 years of experience in pediatrics, selecting, by consensus with team members, the questions that could be considered essential for the objectives of the study and using simple and non-technical language. The study was disseminated through social networks, different pediatric hospitals, early intervention centers, and associations or foundations of children with developmental disorders.

The questionnaire was completed voluntarily by 193 Spanish families.

### 2.5. Data Collection

Once the questionnaire was completed by the family, the data were electronically loaded. Only the researchers of the study had access to identifiable data.

### 2.6. Ethical Considerations

#### 2.6.1. Ethical Approval

The study complies with the Helsinki rules, as well as with the Spanish Law on Personal Data Protection and Guarantee of Digital Rights, of December 2018. The study was also approved by the Ethics and Experimentation Committee of the University of Malaga (Ref No. 75-2020-H).

#### 2.6.2. Informed Consent

The initial contact with the families of children with infantile hemiparesis was conducted online by sending them the questionnaire. They received information about the objectives set regarding the completion of the questionnaire. If they did not have primary education, the informative sheet was read by another person and was completed according to what the family said. Then, they signed an informed consent in which they approved the use of their data for the research purposes and the dissemination of results.

#### 2.6.3. Confidentiality and Privacy

Once coded, the data were stored, thus providing safety to the identity of the participants. To preserve the anonymity of the participants and comply with the precepts of the Law on Personal Data Protection currently in force in Spain, the principal investigator of this project was the only person with access to the dataset, from computers protected by a secure login with a password, which automatically closed after 5 min of inactivity. The information was not disseminated through specific or recognizable personal details. A non-identifiable dataset was shared to enable comparisons with research purposes at national and international levels.

### 2.7. Data Analysis

The collected data were coded, tabulated, and statistically analyzed using Statistical Package SPSS version 25. Continuous data are presented as median and interquartile range (IQR), while categorical data are presented as a number (percentage). Comparison between variables was performed using the Chi-square test for categorical data. Significance was considered at *p* < 0.05. Significantly associated variables with cause of hemiparesis were entered into a multivariate logistic regression analysis to obtain the most significant relationship with the cause of hemiparesis through the relevant odds ratio (OR) calculated with MEDCAL calculator (https://www.medcalc.org/calc/odds_ratio.php, accessed on 28 December 2020) and 95% confidence interval (CI).

## 3. Results

The entire questionnaire (100%) was completed by the mothers of children diagnosed with infantile hemiparesis included in this study (*n* = 193). The most frequent age range of the mothers was 30–40 years (Table 1).

Perinatal stroke (60.1%) was the most common cause of hemiparesis, and the most typical associated disorder was epilepsy (34.2%), with the second largest percentage in this dimension corresponding to an absence of associated disorders (20.7%). The most frequent birth was “no premature” (74.1%) (Table 2). The mean age of diagnosis of infantile hemiparesis was registered at 8 months (IQR: 0–36).

Perinatal stroke (cause of hemiparesis) was significantly associated with epilepsy (OR: 3.24; 95% CI: 1.65–6.35; *p* < 0.001) and attention impairment (OR: 3.56; 95% CI: 1.29–9.82; *p* = 0.01). When the cause of hemiparesis is postnatally, a significant association with cognitive disorder was observed (OR: 20; 95% CI: 7.35–54.45; *p* < 0.001). When the cause of hemiparesis is due to other (other causes) non-perinatal or postnatal aspects, such as surgeries, cancer treatments, or tumors, the greatest significant association was shown with the presence of any associated disorder (OR: 3.71; 95% CI: 1.77–7.76; *p* < 0.001), as shown in Table 3.

Perinatal stroke was associated with moderately premature birth (OR: 2.29; 95% CI: 1.01–5.19; *p* = 0.04), as observed in Table 4. There are not significant associations between postnatal stroke and different births.

In Table 5, a significant association can be observed between perinatal stroke and age of diagnosis between 0 and 3 months (OR: 155; 95% CI: 9.38–2559.91; *p* < 0.001) and 4 and 6 months (OR: 52.26; 95% CI: 3.14–869.6; *p* = 0.006). There is also a significant association with age of diagnosis between 7 and 9 months (OR: 68; 95% CI: 3.18–18.56; *p* < 0.001). In addition, there is a significant association between other causes and age of diagnosis over 12 months (OR: 78.83; 95% CI: 27.90–222.74; *p* < 0.001).

## 4. Discussion

The purpose of this study was to analyze the association between the cause of infantile hemiparesis and different associated factors as birth (premature and non-premature), associated disorders, and age of diagnosis in the Spanish population. The present study was focused on determining what and how conditioning factors are present in children diagnosed with hemiparesis and the association with the cause of hemiparesis.

Infantile hemiparesis with a premature birth predisposes to the development of associated disorders, such as epilepsy [19], as our study also reflects. Therefore, it was observed that the cause of hemiparesis can be related to later appearance of associated disorders. The results show that perinatal stroke was the most common cause of hemiparesis and it had a significant association with epilepsy and attention impairments, and with moderate premature birth. Epilepsy is frequent in children who have suffered a perinatal stroke and it is associated with the initial appearance of convulsions and infantile spasm [20]. Thus, our study corroborates that epilepsy is a disorder associated with infantile hemiparesis, when the cause is perinatal stroke, but also includes the presence of attention deficit. Related to postnatal stroke, it showed a significant association with cognitive impairment, and when the cause of infantile hemiparesis was other (tumors, cancer treatments, or surgery), there was an association with no impairments. It means that the cause of hemiparesis with more associated disorders is perinatal stroke [21]. This is similar to the study of Kirton et al. [21], who observed different problems within perinatal stroke, highlighting the development of behavior disabilities, cognitive impairments, and epilepsy, which is the same as the results obtained in the present study.

These findings are essential to monitor children and to offer adequate treatment to avoid severe sequelae from these associated disorders, such as motor and neuropsychological comorbidities (cognitive deficiencies and emotional/behavioral difficulties) [22,23]. Thus, severe sequelae prevention or treatment of these associated disorders, with the family’s attention, could be carried out at an early age, since perinatal stroke and early diagnosis had a significant association between 0 and 6 months of age, and 7 and 9 months of age with postnatal stroke and more than 12 months with other causes of infantile hemiparesis.

The average age at the time of diagnosis of CP varies depending on the country and environment, ranging from 12 to 27 months [24,25]. In the study of Patel et al. [26], it was found that in most clinical settings, CP is more reliably recognized by 2 years of age. The age of diagnosis of hemiparesis in our study was 8 months, which suggests that there could be early diagnoses in Spain. Therefore, having an early diagnosis would allow for an early referral to initiate the intervention at an early stage. Early diagnosis and initiation of the intervention provide the best potential to improve results in the long term, which is a strength of the current practice [27]. It was demonstrated that correct management of the ketogenic diet in pediatric patients with drug-resistant epilepsy is important from the beginning to avoid side effects [28]. Thus, initiation and maintenance of treatment are the result of concomitant efforts of pediatric neurologists, dieticians, families, and other caregivers. Then, it has also been suggested that different kinds of e-health applications should be used simultaneously, as complementary resources, to improve epileptic patient outcomes in management of the ketogenic diet [29]. Preliminary studies on early therapy in perinatal stroke (e-TIPS) concluded that e-health applications can be used for early diagnosis (0–6 months) [30,31]. It includes modification of the environment, electronic manuals to train the family to carry out early management of the baby, and online follow-up with the therapist to improve the family’s involvement and child’s functioning during development, and increase the use of the affected upper limb [30,31].

Early diagnosis, when communicated respectfully, truthfully, and empathically toward the needs of the parents, results in greater family engagement, enhanced by the individual goals for the child and the family [32,33]. After diagnosis, the families will have the opportunity to search for information about the diagnosis and prognosis of the condition and how to choose the most adequate intervention for their child and for themselves [33,34]. On the other hand, delays in diagnosis can have long-term negative consequences for both the child and the family [27]. They are associated with parental dissatisfaction, stress, and depression and with higher rates of mental health in children and adolescents [15,34].

Study limitations include the lack of validation of the questionnaire directed to families and the lack of motor data. In addition, some important family variables, such as the number of children in the family, the presence of grandparents or other persons taking care of the disabled child, and the role of genetics, were not analyzed. Therefore, this study is considered to be a preliminary or pilot study. The strengths highlight the possibility of detecting associated disorders early. Thus, it would be essential to train families and professionals to manage the possible problems that may arise due to the cause of hemiparesis, offering an early comprehensive approach appropriate to the needs of the family and the child [35].

## 5. Conclusions

The cause of infantile hemiparesis can be associated with the age of diagnosis, birth, and presence of different associated disorders: cognitive, speech, epilepsy, etc. Knowing the possible associations facilitates the prevention of severe sequelae in children and family, implementing an early global therapeutic approach in children with infantile hemiparesis.

## Figures and Tables

**Table 1:** Age of mothers who completed the questionnaire.

Age of Mothers	Years *n*, (%)
<30	49 (25.40)
30–40	110 (57)
40–50	34 (17.60)
>50	0

**Table 2:** Frequencies of the study variables.

Variables	*N* Total = 193 *n* (%)
Cause of hemiparesis	
Prenatal stroke	0 (0)
Perinatal stroke	116 (60.1)
Postnatal stroke	44 (22.8)
Other	33 (17.1)
Associated disorders	
Cognitive	24 (12.4)
Epilepsy	66 (34.2)
Attention	28 (14.5)
Speech	35 (18.1)
None	40 (20.7)
Birth (gestational age)	
Extremely premature	14 (7.3)
Moderately premature	36 (18.7)
No premature	143 (74.1)
Age of diagnosis	
0–3 months	58 (30.05)
4–6 months	29 (15.03)
7–9 months	31 (16.06)
10–12 months	17 (8.61)
>12 months	58 (30.05)

Extremely premature (<28 gestational weeks); Moderately premature (28–37 gestational weeks); No premature (>37 gestational weeks); Perinatal Stroke (from 20 weeks of fetal life to 28 days postnatal life), Postnatal stroke (after 28 days postnatal life, the causes collected were all present etiologies in this time that produce a stroke except tumors, cancer treatments and surgery); other: after 28 days postnatal life, the causes collected were tumors, cancer treatments and surgery produced in that time.

**Table 3:** Association between the cause of infantile hemiparesis and associated disorders.

Cause of Infantile Hemiparesis	Associated Disorders	Odds Ratio (95% CI)	Significance Level
Perinatal stroke	Cognitive	0.009 (0.00–0.16)	0.001 *
	epilepsy	3.24 (1.65–6.35)	<0.001 *
	Attention	3.56 (1.29–9.82)	0.01 *
	Speech	1 (0.47–2.10)	0.99
	none	0.67 (0.33–1.36)	0.28
Postnatal stroke	Cognitive	20 (7.35–54.45)	<0.001 *
	epilepsy	0.48 (0.18–1.2)	0.13
	Attention	0.08 (0.00–1.43)	0.09 *
	Speech	1.63 (0.63–4.21)	0.31
	none	0.05 (0.00–0.91)	0.04 *
Other causes	Cognitive	1.93 (0.79–4.76)	0.15
	epilepsy	0.34 (0.15–0.76)	0.008 *
	Attention	0.60 (0.21–1.67)	0.33
	Speech	0.69 (0.28–1.70)	0.42
	none	3.71 (1.77–7.76)	<0.001 *

OR: odds ratio, CI: Confidence interval, Significance level *: *p* value (<0.05).

**Table 4:** Association between the cause of infantile hemiparesis and birth.

Cause of Infantile Hemiparesis	Birth (Gestational Age)	Odds Ratio (95% CI)	Significance Level
Perinatal stroke	Extremely premature	21.93 (1.29–373.29)	0.03 *
	Moderate premature	2.29 (1.01–5.19)	0.04 *
	No premature	0.24 (0.11–0.53)	<0.001 *
Postnatal stroke	Extremely premature	0.15 (0.01–2.62)	0.19
	Moderate premature	1.56 (0.61–4.02)	0.35
	No premature	1.06 (0.42–2.66)	0.9

OR: odds ratio, CI: Confidence interval, Significance level *: *p* value (<0.05).

**Table 5:** Association between the cause of infantile hemiparesis and age of diagnosis.

Cause of Infantile Hemiparesis	Age of Diagnosis	Odds Ratio (95% CI)	Significance Level
Perinatal stroke	0–3 months	155 (9.38–2559.91)	<0.001 *
	4–6 months	52.26 (3.14–869.66)	0.006 *
	7–9 months	0.42 (0.20–0.90)	0.025 *
	10–12 months	2.5 (0.79–7.9)	0.12
	>12 months	0.004 (0.00–0.03)	<0.001 *
Postnatal stroke	0–3 months	0.03 (0.00–0.54)	0.02 *
	4–6 months	0.08 (0.00–1.37)	0.08 *
	7–9 months	7.68 (3.18–18.56)	<0.001 *
	10–12 months	1.2 (0.32–4.45)	0.78
	>12 months	1.78 (0.77–4.09)	0.17
Other causes	0–3 months	0.01 (0.00–0.25)	0.003 *
	4–6 months	0.04 (0.00–0.66)	0.02 *
	7–9 months	0.47 (0.17–1.30)	0.14
	10–12 months	0.15 (0.02–1.20)	0.07 *
	>12 months	78.83 (27.90–222.74)	<0.001 *

OR: odds ratio, CI: Confidence interval, Significance level *: *p* value (<0.05).

## Appendix A

**Questionnaire Addressed to Families about the Hemiparesis Condition of Their Children from 0 to 3 Years Old**
Dear parents, here are a series of questions that we would like you to answer being objective with what you know about your child and about his condition of infantile hemiparesis.
Date the questionnaire is answered: ____/____/______
Questions
1. Who answers the questionnaire? - Mark this option if you have had difficulty filling in the questionnaire and it had to be answered by another person with their contributions due to the lack of primary studies. - Father. - Mother. - Both: Father and mother. - Other: Who?
2. How old are you?
3. How long have you lived in Spain?
4. Which half of the child’s body is affected?
5. How old is your child right now? Please indicate your child’s date of birth.
6. In relation to the birth, how was it? - Extremely premature (<28 gestational weeks). - Moderately premature (28–37 gestational weeks). - No premature (>37 gestational weeks).
7. At what age was your child diagnosed with infantile hemiparesis?
8. What was the cause of the infantile hemiparesis? - Prenatal stroke (before 20 weeks of fetal life). - Perinatal stroke (20 weeks of fetal life and 28 days postnatal life). - Postnatal stroke (after 28 days postnatal life). - Other. What?
9. Does your child have any disorders associated with infantile hemiparesis? - Epilepsy. - Cognitive impairment. - Attention deficit. - Language alteration. - None. - Other, Which one?
